# Automated Segmentation of Hyperintense Regions in FLAIR MRI Using Deep Learning

**DOI:** 10.18383/j.tom.2016.00166

**Published:** 2016-12

**Authors:** Panagiotis Korfiatis, Timothy L. Kline, Bradley J. Erickson

**Affiliations:** Department of Radiology, Mayo Clinic, Rochester, Minnesota

**Keywords:** FLAIR, convolution, autoencoders, segmentation

## Abstract

We present a deep convolutional neural network application based on autoencoders aimed at segmentation of increased signal regions in fluid-attenuated inversion recovery magnetic resonance imaging images. The convolutional autoencoders were trained on the publicly available Brain Tumor Image Segmentation Benchmark (BRATS) data set, and the accuracy was evaluated on a data set where 3 expert segmentations were available. The simultaneous truth and performance level estimation (STAPLE) algorithm was used to provide the ground truth for comparison, and Dice coefficient, Jaccard coefficient, true positive fraction, and false negative fraction were calculated. The proposed technique was within the interobserver variability with respect to Dice, Jaccard, and true positive fraction. The developed method can be used to produce automatic segmentations of tumor regions corresponding to signal-increased fluid-attenuated inversion recovery regions.

## Introduction

Glioma is the most common primary brain tumor type in adults and has seen little improvement in treatment effectiveness despite being an area of active research. One of the characteristics of gliomas is that it infiltrates the surrounding brain (1). Among gliomas, glioblastomas are the most frequent, are of high grade and aggressive, with median survival time of 14 months (2). Magnetic resonance imaging (MRI) is the most commonly used modality to assess response to therapy because of its superior soft tissue contrast compared with other imaging modalities (3).

Currently, there is significant interest in extracting quantitative imaging biomarkers from MRI including anatomical and functional images that could lead to better patient diagnosis and follow-up. These imaging biomarkers range from texture features to volumetric measurements extracted to predict molecular biomarkers and overall or progression-free survival. The common requirement of all these approaches is the segmentation of tumor from normal brain (4). Automating tumor segmentation could significantly improve treatment planning and follow-up (5), as manual delineation is a time-consuming task because of either artifacts or magnetic field inhomogeneities and it also introduces variability that might reduce the reliability of the image-derived biomarkers.

Brain tumor segmentation is a challenging task, with many researchers and competitors focusing on creating and evaluating newly developed algorithms. In 2012, the Brain Tumor Image Segmentation Benchmark (BRATS) competition (3, 5) was established as part of the MICCAI (International Conference on Medical Image Computing and Computer Assisted Intervention) conference, and since then, it has been the “gold standard” for brain segmentation algorithm testing.

The brain tumor segmentation algorithms commonly described in the literature usually exploit classical image analysis techniques or pattern recognition techniques (6–8) with the more recent approaches using deep convolutional neural networks (9–16).

Each MRI series (image type) reveals different information about the tumor. For instance, T1-weighted (T1w) images after contrast acquisitions reveal information regarding the enhancing part of the tumor, whereas fluid-attenuated inversion recovery (FLAIR) acquisitions capture the edema part of the tumor.

Lesion size in FLAIR images is an important clinical parameter for patient assessment and follow-up. Manual estimation of the volume of the lesions in FLAIR images is time-consuming and highly user-dependent.

Autoencoders have recently been gaining attention for their ability to perform segmentation tasks in medical images (17–19). One advantage of autoencoders against other deep learning approaches is the use of decoders that enables estimation of features suitable for pixel-wise classification (19).

The aim of this paper is to focus on accurate quantification of the abnormal signal areas in the FLAIR acquisitions in patients with glioma. For the purpose of this study, we use convolutional autoencoders trained on the publicly available BRATS data set and evaluate the accuracy on a data set in which 3 expert segmentations were available. Interobserver variability was also measured.

## Materials and Methods

### Data Set

We used both the BRATS 2015 competition data set (N = 186) and a locally collected glioma data set (N = 135). The BRATS scans included 54 low-grade (grades 2–3) and 132 high-grade (grade 4) gliomas. For each tumor, the ground truth was available from the BRATS Web site. All the scans were subjected (by the BRATS authors) to the following preprocessing steps: skull stripping, coregistration to the same anatomical template, and interpolation to 1-mm^3^ voxel resolution.

The locally collected data set consisted of 135 preoperative scans from 135 patients for which signal intensity-increased regions were delineated by 3 experienced image analysts, each with >8 years of experience (user 1, 14 years; user 2, 18 years; and user 3, 12 years). These regions were delineated using MRIcron (20). This data set was used to evaluate the segmentation accuracy of the proposed algorithm (referred to as validation data set).

## Methods

### Preprocessing

For the locally collected data set, skull stripping was performed using an atlas-based technique. This step was not necessary for the BRATS data set, as skull stripping was already performed.

For both the BRATS and local data sets, we applied N4 bias correction (21) followed by Nyúl intensity standardization (22). The N4 bias correction step was used to correct intensity inhomogeneity and artifacts present in MRI acquisitions consisting of low-frequency signals that affect their intensity levels. One challenge with MRI segmentation is the lack of a standard image intensities. The goal of the Nyúl method is to transform the image histograms so that they match the mean histogram determined through training. The algorithms seek to match histograms at certain percentiles.

### Autoencoders

**Figure 1.**
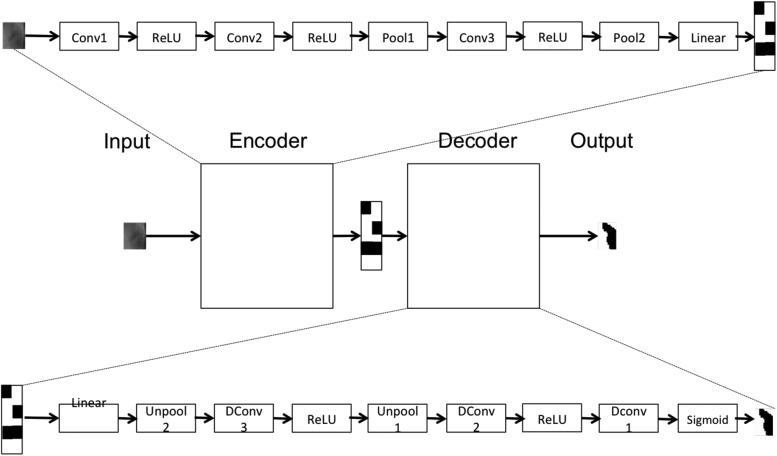
The overall architecture of the developed convolutional autoencoder. Tumor regions were assigned to a value of 1, whereas surrounding tissues were assigned to a value of 0.

Figure 1 captures the main idea of an autoencoder and its application to image segmentation. The primary concept is that the autoencoder learns how to reconstruct the segmented desired output (namely, the segmentation mask). The encoder layer consists of 7 convolutional layers. The convolutions are used to produce the feature maps. In addition, a rectified-linear nonlinearity is applied followed by max-pooling with a 2 × 2 window. The resulting output is subsampled by a factor of 2. Max-pooling achieves translation invariance, accounting for small spatial shifts. The decoder component consists of a hierarchy of decoders, one corresponding to each encoder. Of these, the appropriate decoders use the max-pooling indices received from the corresponding encoder to perform nonlinear upsampling of their input feature maps. This allows for improved boundary delineation (19). The high decoder output is forwarded to a trainable softmax classifier, which independently classifies each pixel. The number of input channels is the number of classes (in our case, tumor or no tumor) and the output of the sigmoid classifier is a 2-channel image of probabilities. The predicted segmentation corresponds to the class with maximum probability at each pixel.

The autoencoder was trained in 100 (out of 132) patients with high-grade glioma included in the BRATS data set and tested on the 32 remaining patients. The training set was used to compute the autoencoder's parameters (such as learning rate), and the smaller one was used as a testing data set to ensure that no overfitting occurred. The pixels with *P* > 0.5 were assigned to the abnormal FLAIR category. All small regions consisting of ≤10 voxels were removed.

**Figure 2.**
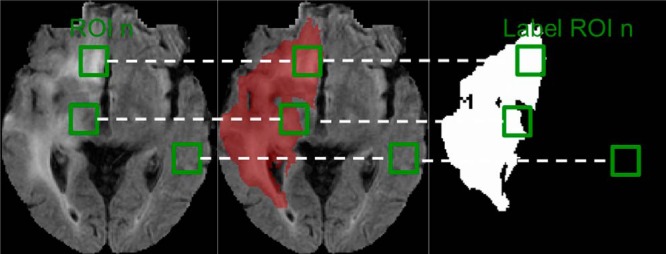
Fluid-attenuated inversion recovery (FLAIR) magnetic resonance imaging (MRI) of a patient with high-grade glioma originating from the Brain Tumor Image Segmentation Benchmark (BRATS) competition data set (Left panel). The red overlay is the ground-truth segmentation highlighting the tumor region (Middle panel). A binary image containing the ground-truth segmentation to depict the different binary patterns contained in the regions of interest (ROIs) considered (Right panel). Green boxes are example ROIs used during the training and testing phases of the autoencoder.

From the extracted region of interest (ROI), a total of 600 000 ROIs were randomly selected. For the purpose of training, ROIs of size 10 × 10 were evaluated (Figure 2).

### Segmentation Evaluation

We applied the simultaneous truth and performance level estimation (STAPLE) (23) method to the local data sets to obtain a probabilistic estimate of the actual ground-truth segmentation incorporating the segmentation data from multiple observers. The STAPLE method assessed the 3 tracings to construct the ground-truth segmentation. To evaluate the proposed algorithm, the following segmentation metrics were calculated: the Dice Coefficient (ie, the similarity index), the Jaccard coefficient (ie, the overlap ratio), and the false positive and false negative fractions. The Dice and Jaccard coefficient values range between 0 and 1, with values closer to 1 indicating closer similarity. Box plot distributions were also calculated. To assess the segmentation results obtained from all the methods considered, the χ^2^ test was performed. For each of the quantitative measures considered in this study, the following 7 first-order statistics were estimated: mean, standard deviation (SD), maximum value (Max), minimum value (Min), median, first quartile (Q1), and third quartile (Q3).

## Results

Comparison between the proposed method and the 3 manual segmentations available against the STAPLE algorithm is shown in Table 1. The statistical differences between the methods is presented in Table 2. Compared with STAPLE, user 1 achieved the best results with respect to all the metrics used. User 3 was the worst performing among all users. The proposed system achieved a Jaccard coefficient of 0.758 and ranked third (within the interobserver agreement) with respect to all measures considered. No statistically significant difference was observed between the proposed system results and those from users 1 and 2 besides the false positive fraction (FPF) metric. User 3 was significantly different for all the metrics besides the true positive fraction metric.

**Table 1.** Comparison Between Proposed Method and 3 Manual Segmentations Available Against STAPLE Algorithm

Measure	Statistic	User 1	User 2	User 3	Proposed
Jaccard	Mean	0.923	0.840	0.758	0.785
	SD	0.051	0.077	0.057	0.095
	Max	1.000	1.000	0.865	0.917
	Min	0.760	0.550	0.649	0.458
	Median	0.931	0.856	0.747	0.821
	Q1	0.901	0.815	0.711	0.729
	Q3	0.957	0.879	0.809	0.849
Dice	Mean	0.959	0.911	0.861	0.876
	SD	0.029	0.048	0.037	0.066
	Max	1.000	1.000	0.928	0.957
	Min	0.864	0.710	0.787	0.629
	Median	0.964	0.922	0.855	0.901
	Q1	0.948	0.898	0.831	0.843
	Q3	0.978	0.935	0.895	0.919
FPF	Mean	0.079	0.198	0.190	0.291
	SD	0.055	0.135	0.111	0.210
	Max	0.253	0.819	0.460	1.181
	Min	0.000	0.000	0.020	0.090
	Median	0.070	0.164	0.169	0.219
	Q1	0.044	0.136	0.100	0.172
	Q3	0.101	0.227	0.275	0.370
TPF	Mean	0.993	0.996	0.899	0.995
	SD	0.032	0.015	0.062	0.016
	Max	1.000	1.000	0.994	1.000
	Min	0.793	0.923	0.720	0.931
	Median	1.000	1.000	0.895	1.000
	Q1	1.000	1.000	0.860	1.000
	Q3	1.000	1.000	0.956	1.000

Abbreviations: FPF false positive fraction; TPF true positive fraction.

**Table 2.** Statistical Comparison Between Proposed Algorithm and 3 Manual Segmentations Against STAPLE Ground Truth

	User 1	User 2	User 3	Proposed
User 1				
Jaccard		<0.001	0.002	<0.001
Dice	—	<0.001	0.004	<0.001
FPF		<0.001	0.011	0.04
TPF		<0.001	0.625	<0.001
User 2				
Jaccard			0.002	<0.001
Dice	—	—	0.004	<0.001
FPF			0.012	0.003
TPF			0.626	<0.001
User 3				
Jaccard				0.093
Dice	—	—	—	0.173
FPF				0.004
TPF				<0.001
Proposed				
Jaccard				
Dice	—	—	—	—
FPF				
TPF				

Abbreviations: FPF false positive fraction; TPF true positive fraction.

There is high interobserver variability for the manual delineation task compared with the STAPLE-derived ground truth, particularly when comparing user 3 to users 1 and 2. In terms of the FPF index, our method performs the worst. As depicted in Figure 6, this can be seen in the false positive segmented regions mostly belonging to the skull region.

**Figure 3.**
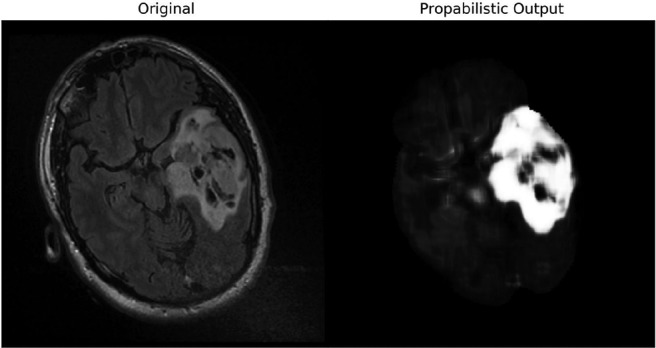
Probabilistic output of the proposed system for a case in our validation data set. As depicted, tumor areas appear to be brighter than surrounding tissue (Right panel).

Figure 3 captures the probabilistic output generated from the autoencoder for an input image.

**Figure 4.**
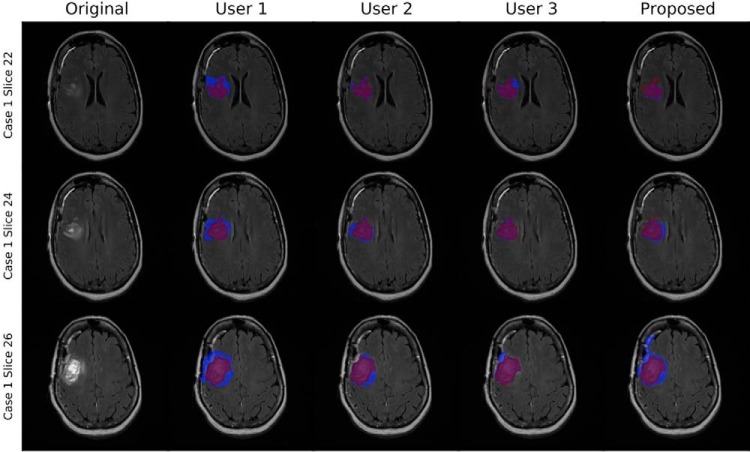
Visual examples of the results of the proposed software compared to the ground truth created using simultaneous truth and performance level estimation (STAPLE) originating in 3 representative sections from 1 subject in the test data set. Blue overlays correspond to oversegmentation regions (nonbrain regions included in the tumor segmentation), whereas red corresponds to undersegmentation (tumor regions not included by the proposed algorithm).

**Figure 5.**
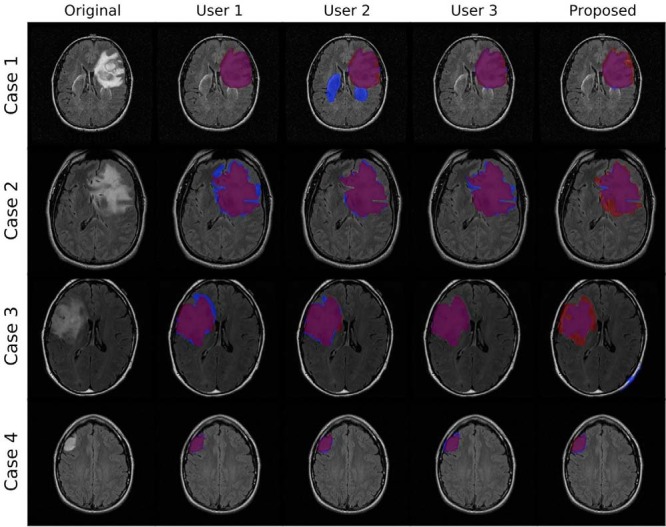
Visual examples of the results of the proposed software compared with the ground truth created using STAPLE originating from 5 different subjects in the test data set. Blue overlays correspond to oversegmentation regions (nonbrain regions included in the tumor segmentation), whereas red corresponds to undersegmentation (tumor regions not included by the proposed algorithm).

Figures 4 and 5 depict representative examples of the algorithm output and its comparison with the 3 users and the STAPLE segmentation.

Figure 5 depicts results on 4 different subjects from the validation data set. In cases 1 and 2, we can observe differences between users 1 and 2 and the way they create the data set. In cases 3 and 4, we can observe undersegmentation resulting from the proposed algorithm.

**Figure 6.**
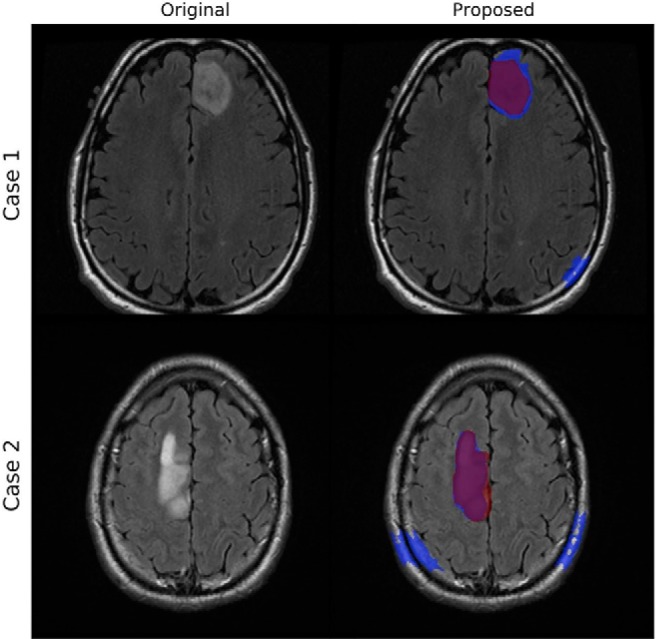
Visual examples of errors in the proposed software compared with the ground truth created using STAPLE. Blue overlays correspond to oversegmentation regions (nonbrain regions included in the tumor segmentation), whereas red corresponds to undersegmentation (tumor regions not included by the proposed algorithm).

Figure 6 captures false positives observed when using our algorithm. The majority of them can be attributed to undersegmentation errors of the skull stripping algorithm (failure to remove all nonbrain tissues). The false positive segmentation errors are more pronounced in case 2.

## Discussion

In this paper, we present an approach based on convolutional autoencoders that segment regions of increased signal intensity on FLAIR images. We used a publicly available data set to train and test the autoencoders, whereas a different data set originating from our institution was used for validation. The validation data set had all the areas of interest manually delineated by 3 image analysts, and STAPLE used these to create a ground-truth labeling.

Our approach was able to produce segmentation results that fell within the interobserver variability with no statistical difference versus 2/3 users compared with the STAPLES output in terms of the Jaccard, Dice, and true positive fraction measures.

Our method was the worst in terms of the FPF metric (Figure 6). This can be attributed to false positive segmentations occurring in areas where the skull-stripping algorithm failed. The data provided from the BRATS competition were those of skull stripping; thus, when we trained our autoencoder, it was unable to learn the skull patterns. This was problematic, as the locally available data set had some skull-stripping failures where the skull was not always removed. To account for this issue, a morphological operation was applied, which eliminated the small false positive regions.

The results of this study indicate that there is substantial interobserver variability for the manual delineation of the abnormal signal on FLAIR images. User 3 had the largest variation compared with the ground-truth variation from users 1 and 2. The challenge of reproducibly segmenting the increased signal regions has been highlighted recently where a Dice coefficient of 0.69 was reported for total tumor volume segmentation in the BRATS data set (24). Akkus et al. (25) reported a 10% variability between 2 different manual segmentations for tumor boundaries on T2-weighted images. Part of the task complexity is that hyperintensity can be due to nontumor disease (eg, vascular disease) and different users may draw different boundaries because of this. An automated algorithm may help standardize the segmentations and make the information that can be extracted more valuable.

Several proposed systems in the literature evaluate their performance against the BRATS data set. In this paper, BRATS was used to train an algorithm, and the performance was evaluated on a locally created data set where the ground truth was based on 3 users (compared with BRATS where the ground truth corresponds to 1 user). We chose to use BRATS as the training data set because the acquisition protocol of the data was more variable than our local data set. Training the algorithm on a less variable data set would likely have been less successful in generalizing to the BRATS data.

Recently, Menze et al. (8) proposed an algorithm based on a generative probabilistic model aimed at tumor and stroke region segmentations. Their work reported a Dice index mean value of 0.73 ± 0.13 when aiming to segment the complete FLAIR lesion, with a 0.86 ± 0.06 interobserver variability. Kwon et al. (26) reported a Dice index of 0.86 using a similar approach. Cordier et al. (27) proposed an atlas-based approach and reported a Dice index of 0.87. Steed et al. (28) reported an accuracy of 0.84 ± 0.09 for FLAIR hyperintensity volumes in a data set of 30 examinations. Porz et al. (24) applied a publicly available tumor segmentation software to a data set of 25 patients with glioblastoma, and reported a Dice coefficient of 0.80. Juan-Albarracín et al. (29) used a Gaussian hidden Markov Random Field and reported a Dice coefficient of 0.77 for tumor area segmentation. Kamnitsas et al. (16), using convolutional neural network and conditional random field, reported a Dice index of 0.901 for whole tumor segmentation. In addition, in a similar approach to the proposed one, Vaidhya et al. (30) reported a Dice index of 0.814 when applied to the BRATS 215 data set.

Recently, a publicly available software tool called BraTumIA (24) for tumor segmentation was used as a means of calculating a set of proposed quantitative measures revealing a good correlation between manual and automatic quantification of the features. Compared with the proposed approach, BraTumIA requires precontrast T1w, postcontrast T1w, T2-weighted and FLAIR images, making direct comparison with the proposed system challenging.

One advantage of the proposed technique against traditional machine learning algorithms is that the features are learned directly from the images without the need of special feature-extraction techniques. The proposed tool was developed using Python (version 2.7.11, Python Software Foundation, http://www.python.org). The autoencoder was developed with the Keras Python Library (https://github.com/fchollet/keras) and the training and execution of the code were done on an NVIDIA K2 grid card (NVIDIA Inc., Santa Clara, California). The algorithm needed ∼1 hour to segment a 256 × 256 × 150 data set.

One significant source of error in our approach was because of poor skull stripping. The BRATS data had human-confirmed accurate skull stripping; thus, the algorithm had this included in its model. However, we used only fully automated methods, and so the human cleanup of skull stripping was absent and resulted in many nonbrain regions being labeled as tumorous. Our approach could benefit from a more robust skull-stripping algorithm.

## Conclusion

The proposed automated system is indistinguishable from expert-derived segmentations in its ability to perform glioma segmentation. This approach will be useful for alleviating the inherent variability of human-derived tumor delineation, thereby improving the reproducibility of image-derived biomarkers.
